# Role of gonadally synthesized steroid hormones in the colorectal cancer microenvironment

**DOI:** 10.3389/fonc.2023.1323826

**Published:** 2023-12-05

**Authors:** Liu Wenxuan, Li Liu, Lilong Zhang, Zhendong Qiu, Zhongkai Wu, Wenhong Deng

**Affiliations:** Department of General Surgery, Renmin Hospital of Wuhan University, Wuhan, Hubei, China

**Keywords:** estrogens, progesterone, androgens, tumor microenvironment, colorectal cancer

## Abstract

**Objective:**

To understand the relationship between steroid hormones synthesized by the gonads and colorectal cancer as well as its tumor microenvironment, in the expectation of providing new ideas in order to detect and treat colorectal cancer.

**Methods:**

Through reviewing the relevant literature at home and abroad, we summarized that androgens promote the growth of colorectal cancer, and estrogens and progesterone help prevent bowel cancer from developing; these three hormones also have a relevant role in the cellular and other non-cellular components of the tumor microenvironment of colorectal cancer.

**Conclusion:**

The current literature suggests that androgens, estrogens, and progesterone are valuable in diagnosing and treating colorectal cancer, and that androgens promote the growth of colorectal cancer whereas estrogens and progesterone inhibit colorectal cancer, and that, in addition, the receptors associated with them are implicated in the modulation of a variety of cellular components of the microenvironment of colorectal cancer.

## 1 Introduction

Colorectal cancer (CRC) is a global disease that represents a serious threat to the lives and health of people around the world, and is a widespread malignant tumor of the gastrointestinal tract. In accordance with the most recent statistics on cancer, colorectal cancer is the third most common cancer in the world, after lung cancer, and the second most common cause of death (1). The cause of this disease, like other malignant tumors, remains unclear and may be related to a number of precancerous lesions and genetic factors.

Several reports suggest that colorectal cancer is considered a steroid hormone-sensitive tumor (2). Steroid hormones include two major groups of sex hormones and adrenocortical hormones, the main ones synthesized in the gonads are estrogens, androgens and progesterone (3). They are cholesterol derivatives that are fat-soluble, thus, they can traverse across the plasma membrane and attach to intracellular receptors(also known as nuclear receptors, NRs), regulating gene expression. Hormones have a significant impact on the progression of colorectal cancer either through a specific target organ or by regulating a metabolic process (4).

Recently, it has been discovered that the tumor microenvironment plays a crucial part in regulating tumor growth and shaping the tumor’s responsiveness to treatment. The microenvironment of tumor is a multifaceted milieu essential for the sustenance and growth of malignant cells, constituted by neighboring vasculature, immune cells, fibroblasts, bone marrow-derived inflammatory cells, diverse signal biomolecules, and extracellular matrix (5). Steroid hormones synthesized by gonads in tumors are closely associated with the tumor microenvironment, and can have an impact on the tumor microenvironment through the release of cell-signaling molecules, which in turn affect cancer cell growth and development.

In this paper, we will discuss the impacts of steroid hormones synthesized by the gonads on colorectal cancer and its tumor microenvironment in order to improve the understanding of the tumor microenvironment and seek new therapeutic targets.

## 2 Steroid hormones synthesized by the gonads

Androgens are steroid hormones that are synthesized mainly by testicular interstitial cells. Androgen production, mediated by the androgen receptor (AR), is the principal function of androgens (6). AR belongs to the nuclear receptor superfamily and is a ligand-dependent transcription factor. AR is transcriptionally activated mainly through a ligand-dependent manner, and the activating ligands are mainly testosterone and dihydrotestosterone. When activated, Dissociation from heat shock protein 90 (HSP90), translocation to the nucleus, binding to AR on target genes and ultimately induces transcription of the androgen response (7).

Estrogen mainly facilitates the development of female secondary sex characteristics and maturation of sex organs. Natural estrogens are mainly estradiol, estrone and estriol. Estrogen exerts various biological effects by combining with the estrogen receptor (ER) and regulating the expression of a series of downstream genes, which is the classical estrogen signaling pathway (8). Estrogen receptors include the traditional nuclear receptors ERα and ERβ, which mediate the genotypic effects of estrogen, i.e., “genotypic” regulation through the regulation of the transcription of specific target genes, and the membranous G protein-coupled estrogen receptor (GPER), which mediates the rapid nongenotypic effects of estrogen and exerts indirect transcriptional regulation through the second messenger system (9, 10).

Progesterone is a steroid hormone which is synthesized by the placenta, the ovaries and the adrenal glands, progesterone is supposed to refer to the natural hormone, while progesterone is a general term that includes progesterone and synthetic progesterone (11). Progesterone hormone and estrogen are inextricably linked, and progesterone hormone builds on estrogen’s actions (12).

## 3 Hormones and their receptors in colorectal cancer cells

### 3.1 Androgens and their receptors and colorectal cancer

Epidemiologic and experimental studies have shown androgens and their influence in developing colorectal cancer. Testosterone treatment accelerates the proliferation of small intestinal and colon tumor cells, which is inhibited after depopulation of colon tumor cells (13). Exogenous testosterone alters tumor distribution and characteristics and inhibits abnormal epithelial cell proliferation (14). Interaction of androgens with the nerve growth factor receptor NGF receptor affects colorectal cancer cells (15). Radiotherapy (46-50Gy) for rectal cancer was associated with markedly elevated serum FSH and LH and markedly lowered testosterone levels (16). Involving androgens in the TUBB3 pathway opens the door to clinical trials evaluating antiandrogens to increase the efficacy of chemotherapy in male colorectal cancer patients (17). The antiandrogen drug flutamide also inhibits colon tumor cell proliferation (13). Activation of the androgen receptor increases the expression of bone morphogenetic protein (BMP) inhibitors but decreases the expression of BMP4 and Wnt inhibitors in primary stromal cells, which promotes the growth of intestinal organoids.Overall, this study reveals the role of androgens in promoting proliferation and inhibiting differentiation, suggesting that stromal cells constitute the microenvironment of intestinal stem cells, it provides a possible explanation for the high incidence of colorectal cancer in men (18).

ERα and AR proteins were elevated in malignant tumor tissues, and ERβ and progesterone receptor (PR) were significantly decreased. AR expression was highest in male tumor tissues, where AR receptor blockers induced apoptosis and testosterone co-treatment impeded the effect (19). Furthermore, AR has been shown to be expressed at both the mRNA and protein levels on both healthy and cancerous colon mucosa (20). Abdulkader M Albasri et al. examined the androgen receptor status in colon cancer patients using immunohistochemistry and correlating the results with all available clinicopathological parameters that predict prognosis. Patients with higher levels of AR expression had significantly worse survival and AR expression may be a prognostic marker for colorectal cancer (21).

ARA54 is an androgen receptor coactivator that enhances AR-dependent activation of transcription. Hirotoshi Kikuchi et al. demonstrated that ARA54 may be involved in promoting cell cycle progression and cell proliferation through the induction of cell cycle protein D1 (22). The human androgen receptor gene contains a polymorphic CAG repeat region that varies from 8 to 35 repeats in the average population. The length of the repeat sequence is negatively correlated with the trans-activation potential of the receptor (23). Long CAG repeats and negative AR expression were both associated with poor 5-year overall survival in colorectal cancer, according to Rui Huang et al. (24). Androgens are known to be expressed on the mucous membranes of the bowel, AR CAG repeat sequences undergo a variety of unique somatic mutations that primarily result in the case of shorter alleles. Colorectal epithelial cells that carry AR alleles with shorter CAG repeat sequences are more sensitive to androgens and are favored to grow (25). Coactivator-associated arginine methyltransferase 1 (CARM1) functions as a transcriptional co-activator of AR-mediated signaling. Young-Rang Kim et al. found that CARM1 is particularly highly expressed in colorectal cancer through tissue microarrays, which was demonstrated by further investigation using surgery specimens. Transcriptional regulator CARM1 by altering the activity of P53 and NF-κB, especially in colorectal cancer (26).

Shuchen Gu et al. were the first to demonstrated that mAR are primarily expressed in colorectal tumors and showed that their activation induces an anti-tumor response *in vitro* and reduces tumorigenicity *in vivo* (27). Vinculin is a protein that controls cell adhesion and actin reorganization. It is efficiently phosphorylated upon mAR activation. mAR activation inhibits the pro-survival signaling Akt/Bad signal pathway *in vitro* and *in vivo* and blocks colon cancer cell migration by regulating neuregulin signaling and actin reorganization, supporting the potent tumorigenic effects of these receptors (28). Gu et al. went on to discover in colon tumor cells the regulation of FAK/mTOR/p70S6K/PAK1 signaling pathway by testosterone-coupled receptors through activation of mAR, which controls early testosterone-induced actin rearrangement in colorectal cancer cells (29). Then mAR was found to be downregulated by specific testosterone albumin coupling (TAC) to regulate the late expression and/or activity of the oncogenic gene products c-Src, GSK-3β, and β-linker proteins, which promotes a pro-apoptotic response in colorectal tumor cells (30).

In summary, androgens and their receptors promote tumor growth in most cases in colorectal cancer, but the related signaling pathways are still not well understood, so more research is needed into the role of androgens and their receptors in colorectal cancer.

### 3.2 Estrogen and its receptors and colorectal cancer

Estrogen and its receptor are essential for CRC tumorigenesis and progression. Ryuichiro Sato et al. used liquid chromatography-electrospray ionization tandem mass spectrometry (LC-ESI-MS) and immunohistochemistry to show that the STS (steroid sulfatase)/EST (estrogen sulfotransferase) status of the cancerous tissues determines the level of estrogen in the tumors, which proves that estrogen is mainly produced locally via the sulfatase pathway and plays an essential role in the progression of the disease progression (31). Estradiol regulates colorectal cancer stem cell bioactivity and interaction with endothelial cells (32). Estrogen promotes the development of inflammation-associated cancers by impairing mucosal responses to inflammatory injury (33). Nrf2 inhibits macrophage inflammatory responses by blocking pro-inflammatory factor transcription (34), and estradiol inhibits CRC by modulating the Nrf2-related pathway (35) Estrogen further inhibits CRC in the absence of Nrf2 by upregulating ERβ-related alternative pathways (36). Estrogen prevents the sustained growth of COLO-205 human colon cancer cells through induction of apoptosis, reduction of c-myb protein and reduction of transcription of the anti-apoptotic protein bcl-2 (37).

Many studies have shown that estrogen action under physiological and pathophysiological circumstances is mediated by estrogen receptors ERα and ERβ as well as membrane-bound GPER (10). Asmaa Abd ElGhany Abd ElLateef et al. reported that lower levels of ER/PR expression were associated with a more extensive CRC primary tumors and poorer prognosis (38). Nancy L Cho et al. also reported that ERα and ERβ are inhibitory modulators of antigen presenting cell (APC)-dependent colon tumorigenesis (39). Overexpression of ERα upregulated hTNF-α gene expression and down-regulated β-conjugated protein signaling activity, which induced apoptosis and inhibited proliferation of LoVo colon cancer cells (40). Hai-ping Jiang et al. reported that an variant of ERα, ERα46, mediated growth inhibition and apoptosis of human HT-29 colon adenocarcinoma cells when estradiol present (41).

The ERβ gene exceeds the ERα gene in the etiology of colorectal cancer (42). ERβ is also the dominant estrogen receptor in the histologically intact colon. Véronique Giroux reported that ERβ deficiency promotes small intestinal tumorigenesis and suggested that regulation of the TGFβ signaling pathway may be a factor in he protective effect of estrogen on intestinal tumorigenesis (43). Francesco Caiazza et al. found that 17β-estradiol induced ERβ upregulation in colon cancer cells by activating p38/MAPK signal pathway (44). ERβ regulates p65 signaling in colon cells (45). Estradiol regulates miR-135b and mismatch repair gene MMR expression in colorectal cells via ERβ (46). Xian Xu et al. also reported that 17β-estradiol agonists inhibited the ability of human LoVo colorectal cancer cells to replicate and migrate via p53 signal pathway (47). The study conducted by Lu et al. shown that estrogen had a positive impact on mismatch repair and tumor suppression both *in vitro* and *in vivo*. This effect was achieved by the induction of MLH1 expression mediated by ERβ (48). A new ERβ agonist, OSU-ERb-12, has been reported to block tumor progression and limit cancer stem cell (CSC) subpopulations (49). The growth of the MC38 colon cancer line was found to be inhibited by diarylpropionitrile, a specific agonist of ERβ, as reported by Ewelina Motylewska (50).. In a study conducted by Nakayama et al., it was discovered that the combination of an ERβ ligand and TMX (tamoxifen) demonstrated a suppressive impact on colon cancer cells (51). Calycosin targets ER, upregulates PTEN, and blocks the PI3K/Akt signaling pathway to slow the growth of colorectal cancer (52). The tumor suppressor protein known as WFDC3 plays a crucial role in inhibiting the spread of tumors in colorectal cancer. This inhibition is achieved through the promotion of ERβ-mediated transcriptional repression of TGFBR1 (53).

Liu Qiao et al. found epigenetic down-regulation of the GPER to act as a tumor suppressor in colorectal cancer (54). Lorna C Gilligan et al. reported that human colorectal cancers promote estradiol synthesis via GPER stimulation to enhance proliferation (55). Maria Abancens et al. identified a mechanistic role for the G protein-coupled membrane estrogen receptor GPER in preventing CRC progression by selectively reducing the tumorigenic effects of the overactive Wnt/β-linker signaling pathway in CRC (56).

The aforementioned findings demonstrate that estrogen and its associated receptors generally impede the progression of colorectal cancer by means of the Nrf2-related pathway, induction of apoptosis, suppression of c-myb protein, and attenuation of the transcription of the anti-apoptotic protein bcl-2. Additionally, estrogen promotes the up-regulation of hTNF-α gene expression and the down-regulation of β-catenin signaling activity, TGFβ-related signaling pathway, and Wnt/β-collagen signaling pathway. Furthermore, estrogen facilitates the up-regulation of p38/MAPK and p65 signaling while down-regulating the Wnt/β-collagen signaling pathway. Therefore, further studies are necessary to understand estrogen and its corresponding receptors in colorectal cancer cells.

### 3.3 progesterone and its receptors and colorectal cancer

Progesterone is an important naturally occurring sex hormone whose cellular effects are mediated by adsorption on the progesterone receptor and modulating hormone-responsive target genes in several cancer types (57). Zhang Y L et al. first analyzed the progesterone levels of 77 patients with CRC, and immunohistochemistry was conducted to identify the overexpression of progesterone receptor in colorectal cancer. S Singh et al. also discovered that the concentration of progesterone receptor in rectal cancer was significantly greater than that in the comparable normal tissues, demonstrating that the concentration of progesterone receptor mRNA has a strong link between cancer and normal tissues (58). The level of progesterone receptor (PGR) expression in CRC tissues correlates with sex, tumor size, degree of differentiation of the tumors, degree of infiltration of the tumor vasculature, and tumor clinical stage. The prognosis of CRC patients with low PGR expression is worse (59). Luteinizing hormone controls the proliferation, apoptosis, angiogenesis, and autophagy of cancer cells, among other cancer cell characteristics (60). Zhang Y L et al. further showed that progesterone increased the expression of growth arrest and DNA damage-inducible protein GADD45α and activated the JNK pathway. Progesterone exerts a stimulatory effect on the JNK pathway via GADD45α, leading to the inhibition of cell proliferation through the suppression of the cell cycle and the induction of apoptosis, thereby inhibiting the malignant advancement of colorectal cancer (59). ER and PR expression and p53 gene detection and DNA ploidy analysis in colorectal cancer can help in clinical classification and prognostic assessment of colorectal cancer patients (61, 62). The progestin drug medroxyprogesterone acetate (MPA), which causes cell cycle arrest by up-regulating cell surface Fas and FasL, has a significant inhibitory effect and causes human colon carcinoma to undergo apoptosis Live and *in vitro* LS174T cells (63). In women, folic acid helps to maintain endocrine function of the ovary. Wang H C et al. found showed that PGR activation in response to a requirement for folic acid (FA) regulated cell migration and proliferation is universal across all cell lines for cancer (64). Ting et al. employed Transwell invasion experiments to demonstrate that FA effectively decreased the invasive potential of colorectal cancer cell lines, namely COLO-205, LoVo, and HT-29 (65). The proliferation of colon cancer cells was found to be inhibited by the activation of PR with the administration of folic acid (FA) (66). In their study, Kuo et al. provided evidence that FA has the ability to suppress the growth of colorectal cancer cell lines by activating the folate receptorα (FRα)/cSrc/ERK1/2/NFκB/p53 pathway. Additionally, they demonstrated the inhibitory effects of FA on COLO-205 tumor growth in an *in vivo* setting (67). In colorectal adenocarcinoma, the presence of progesterone receptors is partially correlated with the presence of estradiol receptors (12).

17β-estradiol and progesterone monotherapies have the same anticancer effects and enhance the tumor-killing effects of CRC in men through the promotion of androgen deprivation mediated by ERβ and PGR, while also blocking oncogenic pathways regulated by ERα (68).

PAQR3, belonging to the progesterone and adipose Q receptor (PAQR) family, is a geographic regulator involved in the negative regulation of the Ras/Raf/MEK/ERK signaling cascade. Wang X et al. found that PAQR3 deletion significantly exacerbated small bowel survival time and tumor area in APC (Min/+) mice. Next, in SW-480 cells, PAQR3 overexpression was found to inhibit cell proliferation rate, anchorage-independent growth, epidermal growth factor-stimulated ERK phosphorylation, and epidermal growth factor-induced nuclear accumulation of β-connexin, and enhanced these colorectal cancers by PAQR3 knockdown. This series of experiments demonstrated the tumor suppressor activity of PAQR3 in the development of colorectal cancer (69).

In summary, progesterone and its receptor inhibit colorectal carcinogenesis and progression through upregulation of GADD45α to enhance the JNK pathway, inhibit cell cycle, induce apoptosis to inhibit cell proliferation, upregulation of cell-surface Fas and FasL to bring about a stop in the cell cycle, and upregulation of p27 to enhance the folate receptor (FR) α/cSrc/ERK1/2/NFκB/p53, etc., and its related drugs, receptor antagonists, have also demonstrated corresponding efficacy (70), but further understanding of more of the mechanisms of occurrence is needed (**Table 1**).

**Table 1:** Effect of sex steroid hormones secreted by the gonads and their receptors on colorectal cancer.

gonadotropin		Related Pathways	Acts with colorectal cancer cells	Related articles
Androgens and their receptors	mAR	Down-regulation of PI-3K and Akt activity induces p-Bad dephosphorylation/activation of p-Bad	inhibition	(26)
	mAR	Up-regulation of FAK / mTOR / p70S6K / PAK1 signalling pathway	Control of early testosterone-induced actin reorganisation in colon cancer cells	(27)
	mAR	Down-regulation of c-Src, GSK-3β and β-collagen signalling pathway inhibition	facilitation	(28)
Estrogen and its receptors	E2	Up-regulation of ERβ-related pathways in the absence of NRF2	facilitation	(34)
	E2	Induction of apoptosis, reduction of c-myb protein and reduction of transcription of the anti-apoptotic protein bcl-2	inhibition	(35)
	E2	p38/MAPK pathway induces ERβ upregulation	facilitation	(43)
	Estradiol agonists	P53 pathway	inhibition	(46)
	ERα	Up-regulation of hTNF-α gene expression down-regulates β-collagen signalling	facilitation	(39)
	GPER	Down-regulation of the Wnt/β-collagen signalling pathway	facilitation	(54)
Progesterone and its receptors	P	Up-regulation of GADD45α enhances JNK pathway, inhibits cell cycle, induces apoptosis and suppresses cell proliferation	facilitation	(59)
	MPA	Up-regulation of cell surface Fas and FasL causes cell cycle arrest	facilitation	(60)
	FA	Up-regulation of p27 enhances folate receptor (FR) α/cSrc/ERK1/2/NFκB/p53 pathway	facilitation	(65)

mAR, Membrane Androgen Receptors; E2, Estrogen; ERα, Estrogen Receptors α; GPERG, protein estrogen-coupled receptor; MPA, Medroxyprogesterone acetate; FA, Folic acid;.

## 4 Androgens, estrogens, progesterones and their receptors in colorectal cancer TMEs

Androgens, estrogens, progesterone and their receptors have been much studied in some tumor microenvironments such as melanoma (71), breast cancer (72), prostate cancer (73), and recent studies have found their role in colorectal cancer. Estrogen stimulates melanoma growth in mouse models via ERα, biases macrophage polarization towards an immunosuppressive state, and thereby enhances CD8+ T-cell exhaustion function, exhaustion, and resistance to immune checkpoint blockade of ICB (74). Through the activation of stromal ER, which in turn normalizes tumor angiogenesis and adjusts the blood supply to the tumor, Estrogen encourages the proliferation of ER-negative cancer cells, preventing hypoxia and necrosis (72). In fibroblasts connected to breast cancer, the nuclear alternative estrogen receptor GPR30 mediates 17-estradiol-induced gene expression and migration (75).

The coregulator hydrogen peroxide-inducible gene 5 (Hic-5) is a metastable adaptor between focal adhesion and the nucleus of prostate myofibroblast cells, and it is a key mediator of AR signaling specificity and sensitivity (76). The induction of drug resistance in PC cells is facilitated by macrophages through the activation of a signaling cascade involving fibronectin (FN1), integrin α5 (ITGA5), and tyrosine kinase SRC (SRC). This cascade is triggered by the cytokine activin A (77). The prostate cancer tumor microenvironment’s androgen-regulated SPARCL1 prevents metastatic spread (73).

The administration of progesterone has been found to facilitate immunomodulation and foster tumor formation within the mouse mammary gland (78). The involvement of membrane progesterone receptor alpha (mPRα) in the promotion of hypoxia-induced vascular endothelial growth factor production and angiogenesis in lung adenocarcinoma is mediated via the activation of STAT3 signaling pathway (79).

A few studies have been undertaken to reveal the function of three hormones in the tumor microenvironment of colorectal cancer. Simon Milette et al. experimentally found that MDSCs in liver metastases from colorectal cancer patients express TNFR2 and that in TNFR2-/- mice, the reduction of intrahepatic MDSCs coincided with the reduction of Treg accumulation at the metastatic site, further concluding that TNF receptor-2 promotes an immunosuppressive microenvironment in the liver that facilitates the colonization and growth of liver metastases. The study revealed that tamoxifen treatment led to an elevation in the accumulation of cytotoxic T cells in the liver of patients with colorectal cancer LM. Furthermore, the findings indicated that estrogen plays a role in regulating the immunological milieu in the liver, which promotes metastasis (80). Postmenopausal women who undergo estrogen supplementation exhibit a decreased susceptibility to advanced colorectal cancer, Jiang L et al. found that inhibition of endogenous extracellular vesicle production reduced the size of MC38 tumors, increased the density of CD8T cells, and reduced the populations of CD4Foxp3Treg cells, PD-L1 macrophages, and MDSCs in MC38 tumors by immunofluorescence assay, flow cytometry, and other experiments. Transforming growth factor β1 (TGFβ) induces Treg cells, MC38-EVs significantly promote Treg cell induction *in vitro*, E2 reduces the level of TGF-β1 in MC38-EVs and impairs Treg induction in MC38-EVs *in vitro*, which in turn inhibits the growth of mouse colon cancer (81). In a study conducted by Vassiliki Tzelepi and colleagues, the authors observed an upregulation of estrogen signaling components in the microenvironment of colorectal cancer. Specifically, the expression levels of ERβ1, AIB1 (nuclear hormone receptor transcriptional co-activator 1), and TIF2 (transcriptional intermediate factor 2) were found to be increased in cancer-associated myofibroblasts. Furthermore, these elevated expression levels were found to be positively correlated with the progression of the disease (82). The programmed death receptor PD-1 holds significant immunological significance. In their study, Song C H et al. documented that estrogen effectively hinders the growth of MC38 tumors by suppressing the expression of PD-L1 and influencing the populations of cells associated with the tumor (83). Mifepristone, a compound that acts as an antagonist to progesterone receptors, has demonstrated positive effects on lifespan and overall well-being in different mouse models of cancer. These models encompass tumors that lack progesterone receptors. The mechanism behind this improvement involves the inhibition of immunomodulatory proteins that serve as natural killer cells within the tumor microenvironment. The utilization of antagonists targeting the luteinizing hormone receptor represents a potentially innovative approach in the field of immunotherapy, with the aim of combating cancer (70).

In summary, estrogen inhibits colorectal cancer liver metastasis via TNF receptor-2, reverses extracellular vesicle-mediated immunosuppression of the tumor microenvironment on colorectal cancer growth in mice, inhibits colorectal cancer tumor growth via down-regulation of PD-L1 expression and M1 macrophages, and progesterone receptor antagonists inhibit colorectal cancer liver metastasis by modulating NK cells. The current information about the three hormones and myeloid-derived cells, macrophages, T cells, and tumor-associated fibroblasts in colorectal TME is relevant, but the specific roles are not well understood. There have also been few studies related to these three hormones and other tumor microenvironment molecules. Therefore, further studies are needed.

## 5 Summary and prospects

This paper summarizes the relationship between androgens, estrogens and progesterone and colorectal cancer and its microenvironment. On the one hand, there is a new understanding of the mechanism of colorectal cancer development, progression and metastasis, androgen plays a promotional role in colorectal cancer development, while mAR promotes apoptosis of tumor cells; on the other hand, estrogen and progesterone-associated receptors are inhibitory to the progression of colorectal cancer. Although the association between these three elements and colorectal cancer is evident, the specific mechanisms involved are still unclear, particularly concerning the diverse cellular constituents and associated elements inside the tumor microenvironment of colorectal cancer. It is known that the accumulation of MDSCs and Treg cells in colorectal cancer liver metastases is TNFR2-dependent in females; estrogen, by reversing extracellular vesicle-mediated immunity, leads to a significant decrease in the PD-L1+M2-like macrophage, Treg cell, and MDSC populations, and an increase in the cytotoxic CD8+ T cell population increased, thereby suppressing the tumor microenvironment inhibiting the growth of mouse colon cancer; the present study investigates the role of estrogen signaling within the microenvironment of rectal cancer. Specifically, it examines the upregulation of ERβ1, AIB1, and TIF2 expression in cancer-associated myofibroblasts, and its correlation with disease progression. Furthermore, the study explores the inhibitory effect of estrogen on the growth of MC38 tumors, elucidating its mechanism of action through the downregulation of PD-L1 expression and modulation of tumor-associated cell populations; The efficacy of mifepristone, a progesterone receptor antagonist, has been demonstrated in enhancing lifespan and enhancing overall well-being in different spontaneous murine cancer models. This includes tumors that lack progesterone receptors. Mifepristone achieves this by inhibiting immunomodulatory proteins that act as natural killer cells within the tumor microenvironment. Mifepristone treatment for colorectal cancer has been less studied and large-scale controlled studies are needed to confirm its ability to inhibit colorectal cancer development. In particular, mifepristone modulates immune cells such as NK cells in the tumor microenvironment. Tamoxifen is more widely used in the treatment of colorectal cancer compared to mifepristone, but the mechanisms involved also need to be further elucidated.

Current studies have shown that the incidence of colorectal cancer is higher in men than in women, but the molecular mechanism of the gender difference is still unclear, so more in-depth studies are needed. The present review elucidates the relationship between these three hormones and the colorectal microenvironment, but the mechanisms related to other immune cells in the tumor microenvironment have not been elucidated yet, and the clinical application of these three hormones in colorectal cancer is not widespread enough, and the use of the hormones alone, hormone replacement therapy, or hormones in combination with other anti-tumor therapies is yet to be developed with the aim of preventing or restricting the growth of *in-situ* colorectal cancers or preventing their distant metastasis. metastasis. Therefore, the study of these three hormones and their receptors and colorectal cancer is very necessary. It is believed that the role of estrogen, androgens, progesterone, and their receptors in the tumor microenvironment of colorectal cancer and its treatment of CRC has potential.

## Author contributions

WD: Writing – review & editing. WL: Writing – original draft. LL: Writing – original draft. LZ: Writing – original draft. ZQ: Writing – original draft. ZW: Writing – original draft.
